# Microbial functional traits in the hyperaccumulating *Noccaea praecox* rhizobiome are metal-dependent and host-driven

**DOI:** 10.1186/s40793-026-00890-y

**Published:** 2026-04-05

**Authors:** Valentina Bočaj, Paula Pongrac, Matevž Likar

**Affiliations:** 1 Biotechnical Faculty, University of Ljubljana, Ljubljana, 1000 Slovenia; 2 Jožef Stefan Institute, Ljubljana, 1000 Slovenia

**Keywords:** Metalliferous soil, Non-metalliferous soil, Root compartment, Rhizosphere compartment, Metagenomics, Microbial functional traits, Microbial resistome

## Abstract

**Background:**

*Noccaea praecox* is a zinc (Zn), cadmium (Cd), and lead (Pb) hyperaccumulating plant native to the Italian peninsula and Western Balkans, where it occurs naturally in both metalliferous and non-metalliferous soils. In the present study, we investigated the effects of soil metal concentrations and the plant host on microbial functional traits, specifically the resistome (i.e., microbial functions associated with metal tolerance and resistance) in two soil compartments: the roots and rhizosphere of *N. praecox*. For this, we collected four plants from each metalliferous and non-metalliferous site and used a metagenomic sequencing approach to characterise microbial functions from paired root and rhizosphere samples, with three root samples per site obtained due to limited biomass, and four rhizosphere samples.

**Results:**

The compartment was the primary driver of the general microbial functional structure. By contrast, the soil metal concentrations and root compartment significantly shaped the microbial resistome. Functions associated with the cobalt-zinc-cadmium efflux system and copper-transporting P-type ATPase V were significantly enriched at the metalliferous compared to the non-metalliferous site, with log_2_ fold change being 2.62 and 1.72, respectively. Transporters associated with manganese/iron and cobalt/nickel were shaped by the host, regardless of soil metal levels, consistent with host-mediated filtering of microbial functions. Notably, several Zn transporter-related microbial functions associated with the ZIP family were more abundant in the rhizosphere, potentially supporting the plant’s high Zn demand.

**Conclusion:**

Overall, our results demonstrate that both environmental conditions and plant host play interactive roles in shaping the microbial functional potential, with the host sometimes exerting a stronger influence than soil metal content. The enrichment of Zn transporters (Zrt-/Irt-like proteins) in the rhizosphere of the Zn-hyperaccumulating *N. praecox* suggests a specific microbial adaptation that may facilitate Zn uptake. These findings provide new insight into the functional dynamics of plant-microbe interactions that support the *N. praecox* lifestyle.

**Supplementary Information:**

The online version contains supplementary material available at 10.1186/s40793-026-00890-y.

## Introduction

Hyperaccumulating plants, many of which belong to the Brassicaceae family, have evolved a unique set of traits that enable them to (hyper)accumulate exceptionally high concentrations of metal(loid)s, primarily in their leaves, without showing apparent signs of toxicity [1, 2]. Within the Brassicaceae, *Noccaea praecox* (Wulfen) F. K. Mey. has been identified as a Zn, Cd, and Pb hyperaccumulator [3–5], a rare phenomenon, as most hyperaccumulators predominantly (hyper)accumulate only one metal, most often nickel (Ni) [6].

In addition to abiotic environmental factors, plants interact with various biotic factors, especially microorganisms, which can colonise the surfaces and/or tissues of any plant organ. In the rhizosphere, microorganisms can significantly affect metal availability in soil, their uptake by plants, and their translocation within plants [7, 8]. The development of high-throughput sequencing has revolutionised our ability to study microbial communities in situ, providing detailed insights into their diversity, interactions, and functional traits. As a result, our understanding of the plant microbiome and its effects on the host plant, including its role in enhancing tolerance to abiotic stresses [9], has greatly improved. Given that some hyperaccumulators can establish populations in both non-metalliferous and metalliferous soils [10], they can serve as an attractive model for resolving how contrasting environments, particularly differences in soil metal concentrations, influence root-associated microbiomes. Although studies on the taxonomic composition of microbiomes in hyperaccumulating plant species have increased substantially in recent years, their functional traits remain understudied. Yet, these traits are essential for uncovering the mechanisms driving plant-microbe interactions and for understanding their contributions to the hyperaccumulating lifestyle.

All organisms must develop adaptive strategies to survive in metal-enriched environments. High concentrations of metals reduce soil cation exchange capacity, nutrient availability, and organic matter content [11]. In addition, metals induce the production of reactive oxygen species, which can cause DNA damage [12, 13]. Since metals cannot be degraded, microorganisms have evolved various tolerance and resistance mechanisms to persist under such hostile conditions [14]. Although the definitions of tolerance and resistance in bacteria primarily focus on antimicrobial agents, the concepts have also been extended to metals [15]. Tolerance enables microbes to survive transient exposure [16], possibly in an inactive state [17, 18], and involves mechanisms that reduce cell membrane permeability through alterations in fatty acid composition and the activation of efflux pumps [19, 20]. Among these, ATP-binding cassette (ABC) transporters play a central role in metal tolerance [21, 22]. Additionally, molecules such as glutathione and metallothionein, along with DNA repair systems, are crucial components for metal tolerance [23, 24]. Resistance, on the other hand, allows microbes to grow throughout exposure to the agent [16] and includes mechanisms such as P-type ATPases and members of the cation diffusion facilitators (CDF) family, which function as chemiosmotic ion-proton exchangers [25], as well as reductases [26–28] and chelatase [29]. Bacteria have also evolved multimetal resistance systems encoded on plasmids such as pMOL28 and pMOL30 [30]. These mobile genetic elements carry various resistance genes, including those for Zn, Cd, and Pb, and are transmitted via horizontal gene transfer, enabling rapid adaptation to metal stress [28, 30, 31].

Although studies on microbiome functionality in (hyperaccumulating) plants remain limited, some evidence suggests that microbial functional responses are closely linked to metal exposure. In the arsenic (As) hyperaccumulating fern *Pteris vittata* L., As-resistance microbial genes in the roots were the most abundant As-related metabolic genes, with their abundance increasing in response to higher As concentrations in the soil [32]. Similarly, in the roots of Zn and Cd hyperaccumulating *Sedum alfredii* Hance, microbial functions related to membrane transport and energy metabolism were more enriched in the hyperaccumulating genotype compared to the non-hyperaccumulating one [10]. On the other hand, the plant host has a significant impact on the microbial community by releasing bioactive molecules into the rhizosphere [33], thereby attracting specific microorganisms. In a study by Zhang et al. [34], the root-associated bacterial community was primarily influenced by the root compartment rather than contamination level, indicating a strong influence of the plant host. Together, these findings suggest that both the environment and plant-driven selection act in concert to shape the functional traits of the root-associated microbiome. Understanding this interplay is key to uncovering the mechanisms that underlie the hyperaccumulation and plant-microbe co-adaptation in metalliferous soils.

In this study, we investigated microbial functional traits in the rhizobiome of *N. praecox* that occurs naturally at non-metalliferous and metalliferous sites in Slovenia. The aim was to assess the effects of environmental conditions, particularly differences in soil metal concentrations and plant host, on microbial functions. We hypothesised that (i) metal-enriched conditions affect the abundance of genes related to microbial tolerance and resistance to Cd, Zn, and Pb, which are predominant at the metal-enriched site, (ii) microbial tolerance and resistance to other metals follow a similar pattern due to the shared transfer pathways via plasmids, and (iii) the microbiome differs between roots and rhizosphere. To test these hypotheses, we (i) compared the overall microbial functional traits in the two soil compartments (i.e. roots and rhizosphere) in *N. praecox* collected at two sampling sites, and (ii) examined microbial functions specifically associated with metal tolerance and resistance (resistome).

## Methods

### Site description

Samples were collected in spring 2022 at two locations in Slovenia where *N. praecox* occurs naturally: in Lokovec (N 46° 2′ 39.2706″, E 13° 46′ 8.9934″), a non-metalliferous site, and in Žerjav (N 46° 28′ 26.1258″, E 14° 51′ 56.0118″), a highly metalliferous site with a history of mining and smelting activities. Both sites are characterized by Phaeozems soils developed on calcareous rocks and soils exhibit near-neutral pH values (6.8 in Lokovec and 6.9 in Žerjav) and a high base saturation. Calcium is the dominant exchangeable base cation (30.2–52.1 mmol_c_ 100 g^−1^), followed by magnesium (2.9–9.3 mmol_c_ 100 g^−1^) and potassium (0.39–0.61 mmol_c_ 100 g^−1^), while exchangeable sodium is negligible in both locations. The cation exchange capacity is high at both sites, which corresponds with the substantial organic matter content of 17.1% in Lokovec and 20.2% in Žerjav [35]. Nevertheless, both sites differed substantially in their soil metal concentration, with soil in Žerjav containing much higher concentrations (Pb 9,078 ± 1,245 mg kg^−1^; Cd 38 ± 8 mg kg^−1^; Zn 220 ± 28 mg kg^−1^) than soil in Lokovec (Pb 117 ± 23 mg kg^−1^; Cd 1.1 ± 0.04 mg kg^−1^; Zn 10 ± 3 mg kg^−1^) [36].

### Sample collection

At each site, four flowering plants, formally identified as *N. praecox* by Matevž Likar, were randomly collected along with their rhizosphere and the surrounding soil, placed in plastic pots, and transported to the laboratory, where the roots and rhizosphere were separated. The rhizosphere, defined as the soil tightly adhering to the roots, was obtained by gently shaking the plant [37] and comprised microbes not in direct contact with the root surface. Roots, containing both endophytic and epiphytic microorganisms, were thoroughly washed with tap water, followed by autoclaved distilled water, and finally blotted dry with paper towels. Sample handling was performed with sterilized equipment, which was replaced between each sample to prevent cross-contamination. All samples were stored at −80 °C until further use.

### Metagenomics

Whole-microbial DNA from the rhizosphere (*N* = 4) and total genomic DNA from plant roots (*N* = 3, due to insufficient root biomass of one sample) were extracted using the DNeasy^®^ PowerSoil^®^ Pro Kit (Qiagen) and GenElute™ Plant Genomic DNA Miniprep Kit (Sigma Aldrich), respectively, following the manufacturer’s instructions. The 14 DNA samples were stored at −80 °C until sequencing. The DNA quality control and shotgun metagenomic sequencing were performed by Macrogen company using the TruSeq DNA kit (Illumina) on the Illumina HiSeqX platform (2 × 150 bp) according to the manufacturer’s instructions. On average, sequencing yielded 29,776,694 ± 6,228,348 reads/sample.

Shotgun sequencing data were analyzed with the SqueezeMeta pipeline (v1.7.2), a fully automated pipeline for metagenomics that covers all steps of the analysis [38]. In short, adapter removal, quality filtering, and trimming of the reads were done by Trimmomatic [39]. The high-quality reads were assembled into contigs using MEGAHIT v1.2.9 [40] via the sequential mode. Prinseq was used to remove short contigs (200 bp) and determine the contig statistics [41]. The contigs were subjected to gene prediction using Prodigal software v2.6.3, which was employed to retrieve the corresponding amino acid sequences [42]. Diamond v2.1.9 [43] was used to search for similarity between the NCBI nr database [44]. For functional assignment, Diamond was also used to compare gene sequences against the Kyoto Encyclopedia of Genes and Genomes (KEGG) Orthology database [45].

### Bioinformatics and statistical analysis

All statistical analyses were performed in R (v4.4.2). The datasets analysed during the current study are available in the Zenodo repository, 10.5281/zenodo.15639780. The sequencing quality monitoring (SQM) tools (SQMtools, v1.7.2) package in R [46] was used to analyze functional profiling data generated from the SqueezeMeta pipeline.

For resistome, microbial functions associated with metal tolerance and resistance were assigned by combining KEGG database annotations and literature data (Table S1). Genes were classified into two functional categories: metal tolerance and metal resistance, as described by Muñoz-García et al. [15]. Functions linked to efflux mechanisms—primarily membrane transporter proteins and efflux pumps such as ABC transporters—were designated as tolerance-related [21, 47–49]. By contrast, functions related to resistance, such as functions involved in the enzymatic transformation of metals, P-type ATPases implicated in detoxification processes [50], and proteins with oxidoreductive activity, were classified as resistance-related functions. In addition, functions previously defined as resistance-related in the KEGG database were also included in this category. To ensure reproducibility, we applied a hierarchical classification: (i) resistance functions were defined as those involved in active detoxification and high-specificity control of metal stress. This category included genes encoding enzymatic transformation processes, such as reduction, oxidation, methylation, cofactor assembly, and phosphatase activity, as well as any gene explicitly annotated as a “resistance” factor in KEGG, including pumps or transporters (e.g., czc, ter, cop, ars, mer). In addition, regulatory proteins specifically associated with resistance operons (e.g., zur, merR, nikR), excluding regulators described solely as efflux controllers, and P-type ATPases implicated in active detoxification were classified as resistance; (ii) tolerance functions were defined as those associated with general metal homeostasis, export, and cellular maintenance. This category included efflux mechanisms such as membrane transporters and efflux pumps (e.g., RND and HME families) not explicitly labelled as resistance proteins (e.g., chrA, fieF), general transport systems including permeases, MFS transporters, and standard ABC transporters unless identified as high-affinity scavenging systems, proteins involved in auxiliary maintenance related to metal stress (such as peptidases, proteases, and sequestration proteins, excluding specific cofactor storage), and regulatory proteins explicitly described as controlling efflux systems (e.g., cueR); (iii) manual override: in cases where literature explicitly supports a change of the category. The tolerance and resistance classified functions based on this hierarchical classification are presented in Table S1.

The microbial functional traits, including the resistome, were analysed using the phyloseq package (v1.50.0) [51], which integrates counts, taxonomic, and sample information data for downstream analyses. To account for varying library sizes while maintaining the statistical properties of the data and preserving the sparsity pattern, samples were normalized via scaling to the median sequencing depth. This normalized matrix was used for beta-diversity analysis; specifically, non-metric multidimensional scaling (nMDS) was performed based on Bray-Curtis dissimilarities, and permutational multivariate analysis of variance (PerMANOVA) was calculated using *adonis* function in the vegan (v.2.6-10) package, with p-value < 0.05 considered statistically significant.

To compare the resistome between the two sampling sites, a Wilcoxon Rank-Sum test was applied to the same normalized matrix as used in beta-diversity tests. Multiple testing was accounted for using the Benjamini-Hochberg correction, with functions considered statistically significant at p-value *<* 0.05. The use of a rank-based Wilcoxon Rank-Sum test ensured robust results across scaling methods while accounting for the non-normal distributions typical of functional counts. To test the robustness of the results, we verified that the rank structure remained stable across different normalization approaches. The results showed that the ranks were stable, yielding identical p-values.

For high-resolution identification of specific differentially abundant resistome-associated functions, the DESeq2 (v1.46.0) package was used on raw count data. By utilizing a negative binomial framework and internal size-factor normalization (Median of Ratios), DESeq2 accounts for compositional biases, library size variation, and the overdispersion typical of functional count data. Significance thresholds were set at an adjusted p-value (padj) < 0.01 and an absolute log_2_ fold change (|log_2_FC|) > 1.

The co-occurrence networks were constructed separately for positive and negative correlations using the circlize package (v. 0.4.16). To assess the strength of associations between microbial functions, Spearman’s correlation coefficient was calculated. For the network representing positive correlations, only associations with a Spearman’s rho greater than 0.8 were included. In contrast, the negative co-occurrence network displays microbial resistome functions with a rho value below − 0.8.

Results were visualized with ggplot2 (v3.5.1), cowplot (v.1.1.3) and flextable (v.0.9.7) packages, while the circlize package (v0.4.16) was used for visualisation of correlation plots.

## Results

### Microbial functional traits

Shotgun metagenomic sequencing identified a total of 8,901 microbial functions. For overview analyses of the general functional profile, we retained 748 microbial functions with read counts exceeding 30,000 across all root and rhizosphere samples of *N. praecox* from both sampling sites, in order to focus on the most robust and highly abundant functions for visualization.

**Fig. 1:**
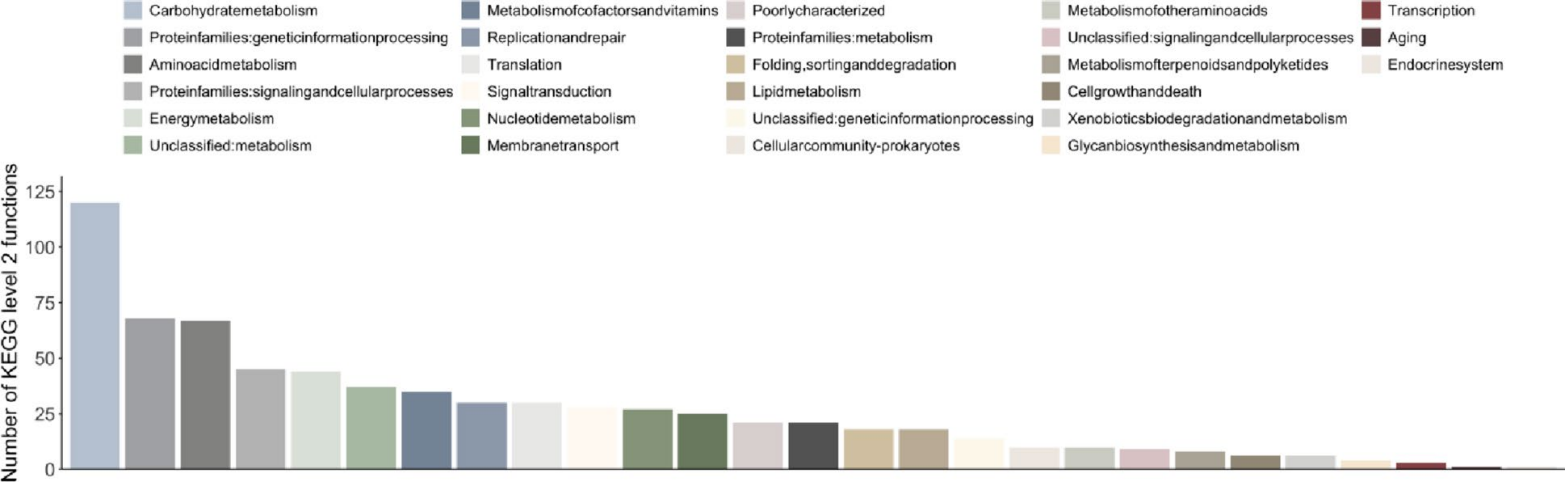
Number of reads for each KEGG level 2 group microbial functions in the root and rhizosphere compartments of *Noccaea praecox* from both non-metalliferous and metalliferous sites

Most microbial functions occurring in the roots and the rhizosphere of *N. praecox* at both sampling sites were associated with KEGG level 2 Carbohydrate metabolism, followed by Protein families: genetic information processing and Amino acid metabolism (Fig. 1). Other KEGG level 2 categories were considerably less abundant in the rhizobiome of *N. praecox*.

Nine KEGG level 2 functions were significantly different between locations, namely Folding, sorting and degradation, Protein families: genetic information processing, Protein families: metabolism, Signal transduction, Transcription, Unclassified: genetic information processing, Environmental adaptation, Metabolism of terpenoids and polyketides, and Transport and catabolism (Table S2). The majority of these functions were more abundant on the metalliferous site (Žerjav) than at the non-metalliferous site (Lokovec), except Unclassified: genetic information processing, which was more abundant on the non-metalliferous site (Lokovec) than on the metalliferous site (Žerjav).

**Fig. 2:**
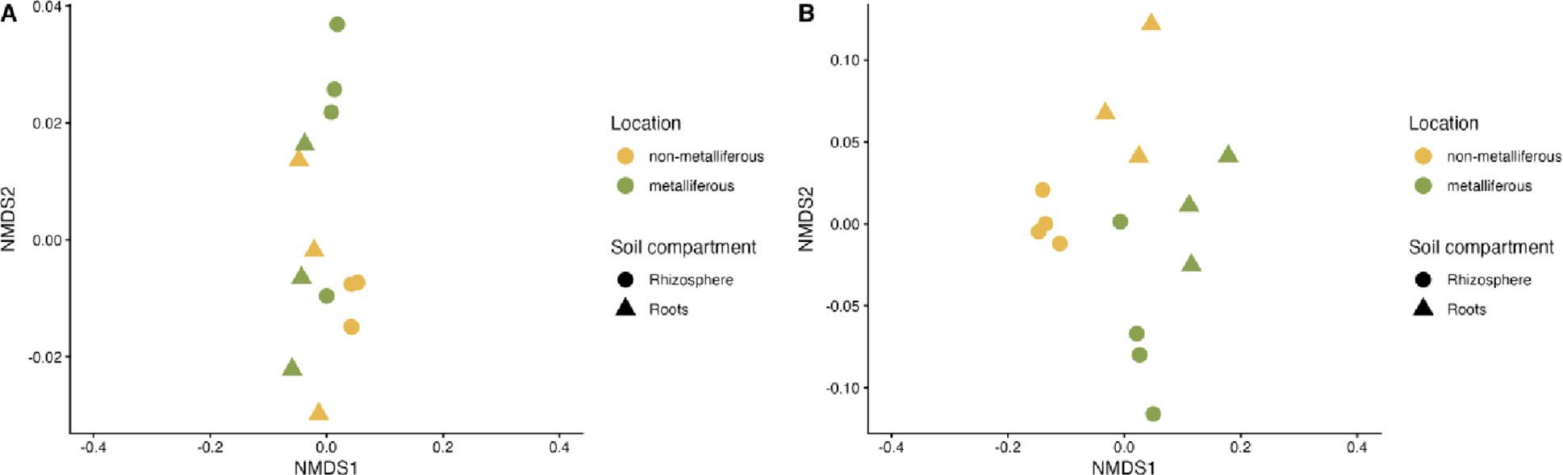
Non-metric multidimensional scaling (NMDS) analysis of the (**A**) microbial functional traits and (**B**) microbial functions related to metal tolerance and resistance (i.e., the resistome) in the root and rhizosphere compartments of *Noccaea praecox* from the non-metalliferous and metalliferous sites. Stress values: 0.0312 (**A**) and 0.0840 (**B**)

Non-metric multidimensional scaling (NMDS) revealed that microbial functions in the roots of *N. praecox* were largely similar between the two sampling sites, while the rhizosphere showed clear differentiation between the two *N. praecox* populations (Fig. 2A).

PerMANOVA analysis confirmed the soil compartment (roots vs. rhizosphere) as a significant factor influencing the microbial functional traits of *N. praecox* (Table S3). While the sampling site alone was not significantly affecting the microbial functional traits, its interaction with the soil compartment did (p-value < 0.05), indicating a context-dependent effect of location.

### Microbial functions associated with metal tolerance and resistance—the resistome

The microbial resistome, comprising microbial functions associated with metal tolerance and resistance, included 114 functions identified in the roots and the rhizosphere of *N. praecox* from both sampling sites (Table S1).

Of 114 functions detected in the microbial resistome of *N. praecox*, 59 were related to metal tolerance and 55 to metal resistance. Among the tolerance functions, the majority of reads were associated with Ni (54.2%, i.e., 818,275 reads), followed by iron (Fe) (17%, i.e., 256,993 reads), and molybdenum (Mo; 8.1%, i.e., 122,730 reads). The most abundant functions (relative abundance > 5%) were related to Ni tolerance and included: (i) peptide/nickel transport system substrate-binding protein, ABC.PE.S (11.5%), (ii) peptide/nickel transport system ATP-binding protein, *ddpD* (8.23%), (iii) peptide/nickel transport system permease protein, ABC.PE.P1 (7.97%), (iv) peptide/nickel transport system permease protein, ABC.PE.P (7.76%) and (v) peptide/nickel transport system ATP-binding protein, *ddpF* (6.39%). Other tolerance functions were related to Fe, Mo, copper (Cu), cobalt (Co), Zn, Cd, chromium (Cr), manganese (Mn), heavy metals (HM), gold (Au), and silver (Ag), each with relative abundance < 3%.

Among the resistance functions, most reads were associated with Cu (25.2%, 111,542 reads), Co (21.9%, 96,667 reads), and Co/Zn/Cd (18.4%, 81,282). The most abundant resistance functions included: (i) P-type Cu+ transporter associated with Cu resistance, *copA*, *ctpA*, *ATP7* (4.81%), (ii) cobalt-zinc-cadmium resistance protein CzcA associated with Co/Zn/Cd resistance, CzcCA (3.6%), (iii) cobalt chelatase CobN associated with Co resistance, *cobN* (1.93%), and (iv) tellurite resistance protein TerC associated with tellurium (Te) resistance, *terC* (1.18%). Other resistant functions were associated with Mn, Mo, Ni, As, mercury (Hg), and Fe, each with relative abundance < 1%.

NMDS of the microbial resistome also showed differences between the two populations (Fig. 2B), along with a clear separation of the roots and rhizosphere compartments. These differences were statistically supported by PerMANOVA analysis, which identified both compartment (p-value < 0.05) and location (p-value < 0.05) as significant factors influencing the microbial resistome of *N. praecox* (Table S4). Furthermore, a significant interaction between the two soil compartments and location (p-value < 0.05) indicated a combined effect of the compartment and environmental context on the functional structure of the resistome.

**Fig. 3:**
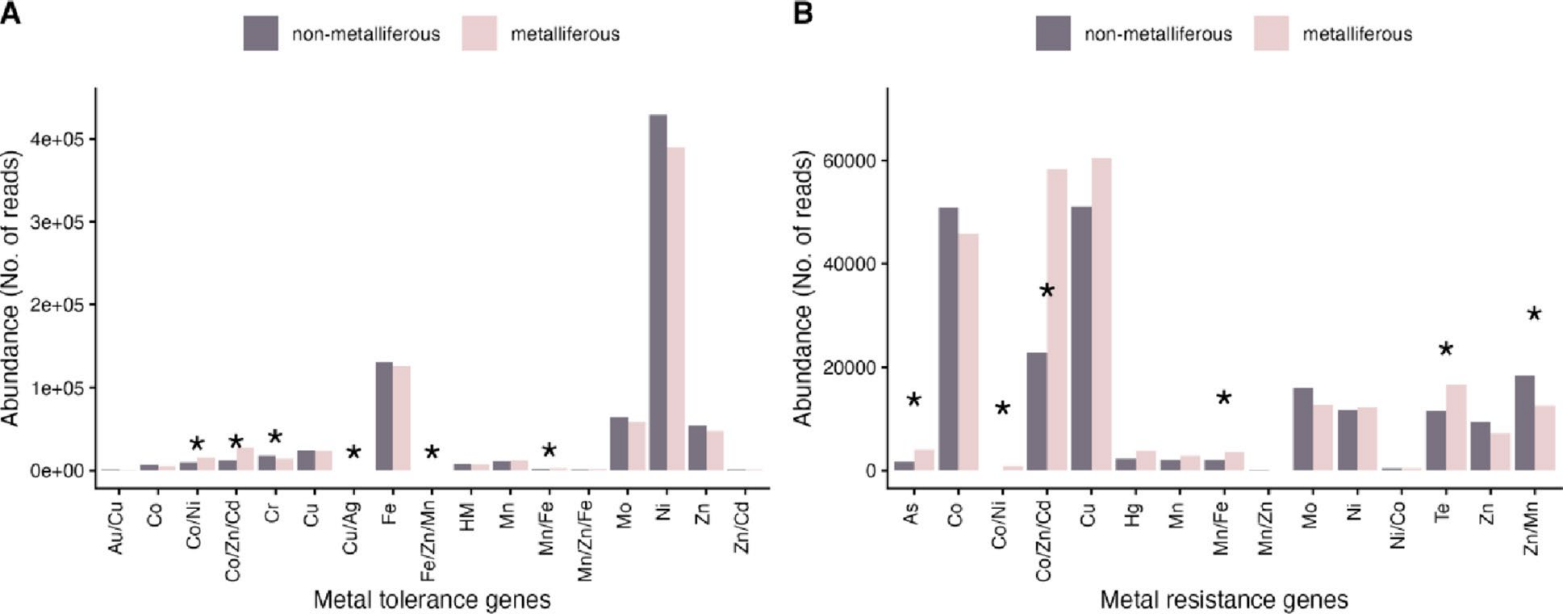
Abundances of functions associated with tolerance (**A**) or resistance (**B**) to specific metal(loid)s in the rhizobiome of hyperaccumulating *Noccaea praecox*. Asterisks (*) depict statistically significant differences in the Wilcoxon test between non-metalliferous and metalliferous sites at *p* < 0.05

The Wilcoxon test further confirmed statistically significant differences in the microbial resistome of *N. praecox* between the non-metalliferous and metalliferous site (Fig. 3). Specifically, significant differences in microbial functions related to metal tolerance were observed for the following metals or metal combinations: Co/Ni, Co/Zn/Cd, Cr, Cu/Ag, Fe/Zn/Mn and Mn/Fe (Fig. 3A). Functions associated with Co/Ni, Co/Zn/Cd and Mn/Fe were more abundant at the metalliferous site, whereas those related to Cr were more abundant in Lokovec. Microbial functions related to Cu/Ag were found only in Žerjav, whereas Fe/Zn/Mn were found only at the non-metalliferous site. By contrast, metal resistance functions differed significantly for: As, Co/Ni, Co/Zn/Cd, Mn/Fe, Te, and Zn/Mn (Fig. 3B), all of which, except functions related to resistance to Zn/Mn, were more abundant at the metalliferous than at the non-metalliferous site.

**Fig. 4:**
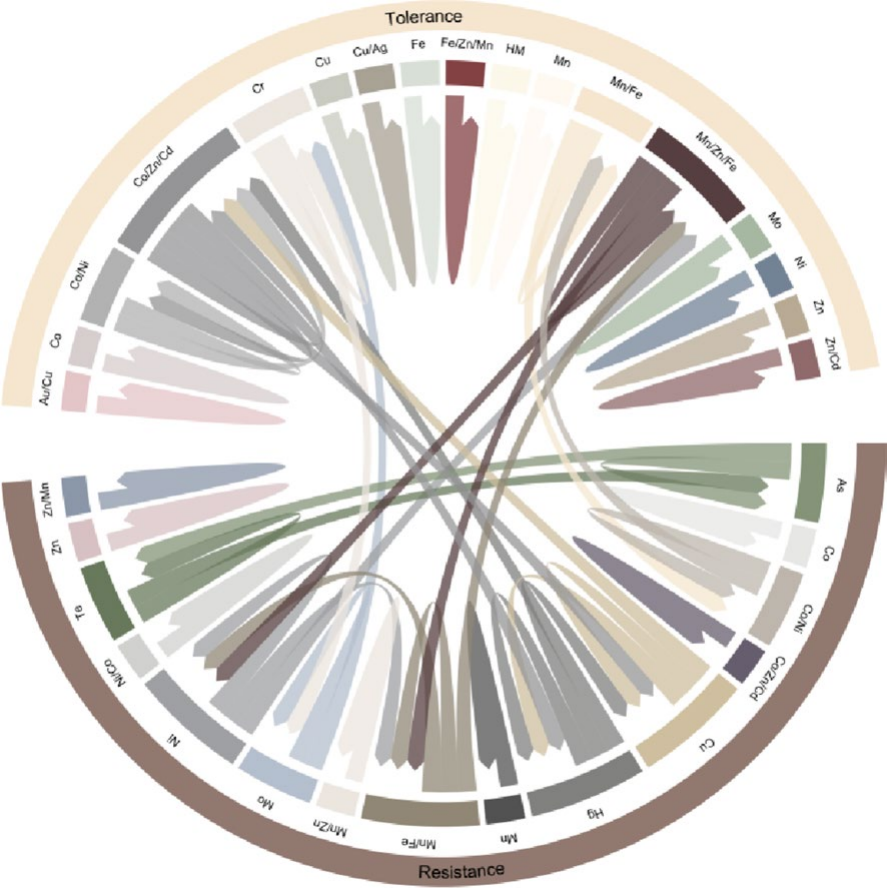
Co-occurrence network for microbial resistome in *N. praecox* rhizobiome with Spearman’s rho > 0.8. Metal(loid)s represent functions associated with either resistance or tolerance to the specific metal(loid)

Strong positive correlations (Spearman’s rho > 0.8) were observed in the microbial resistome co-occurrence network, both among functions associated with metal tolerance and resistance and between them (Fig. 4). The highest number of positive correlations was observed for microbial functions associated to metal tolerance for Co/Zn/Cd and Mn/Zn/Fe, and for metal resistance to Cu, Hg, Mn/Fe, and Ni.

Among the 17 metal-tolerance-related functions, six revealed strong positive correlations with other functions, with over 29% exhibiting strong associations with resistance-related functions. Similarly, eight out of 15 metal resistance-related functions were strongly correlated with other functions, of which 40% were significantly correlated with tolerance-related functions.

**Fig. 5:**
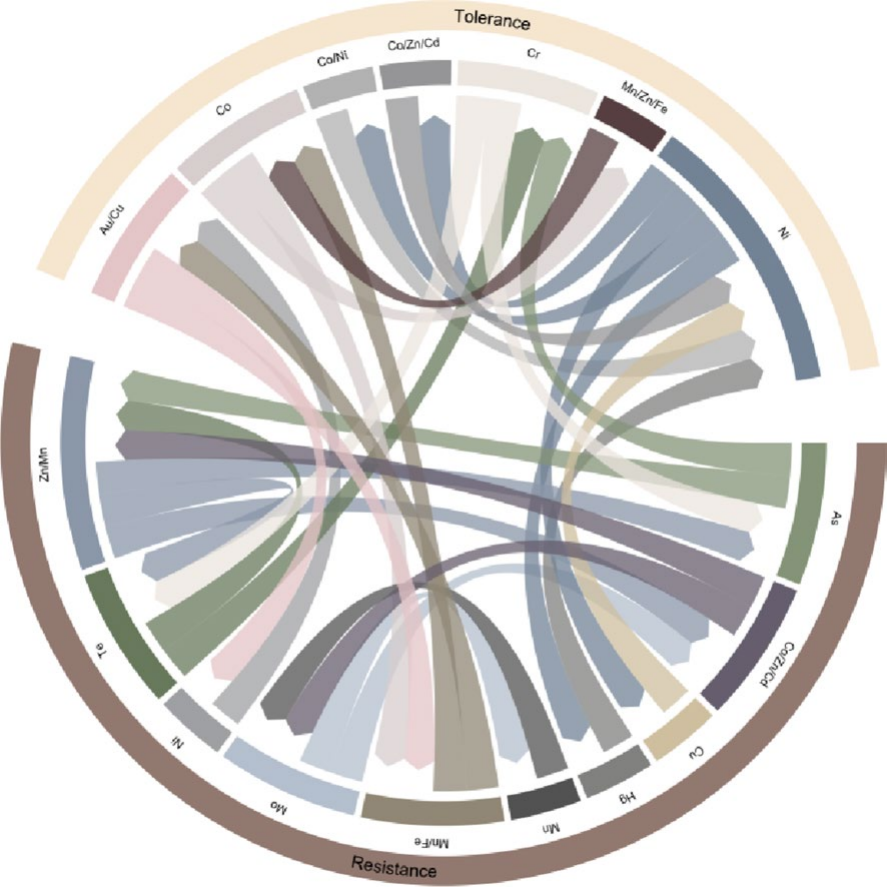
Co-occurrence network for microbial resistome in *N. praecox* rhizobiome with Spearman’s rho < 0.8. Metals represent functions associated with either resistance or tolerance to the specific metal

The co-occurrence network also revealed strong negative associations among microbial resistome functions, with Spearman’s rho values less than − 0.8. (Fig. 5).

Most negative correlations were observed for microbial functions associated with Ni tolerance and Zn/Mn resistance. All microbial functions formed at least one strong negative correlation with other functions.

DESeq2 analysis of the microbial resistome in the root and rhizosphere compartments of *N. praecox* revealed 14 differentially abundant and statistically significant microbial functions between the metalliferous and non-metalliferous sites (Table 1). The functions most significantly enriched at the metalliferous site included: (i) outer membrane protein, cobalt-zinc-cadmium efflux system, (ii) copper-transporting P-type ATPase V, (iii) arsenical resistance protein ArsH, and (iv) tellurium resistance protein TerD. In contrast, functions significantly enriched at the non-metalliferous site were: (i) manganese transport system substrate-binding protein, (ii) uncharacterized zinc-type alcohol dehydrogenase-like protein, (iii) cobalt-precorrin-5B (C1)-methyltransferase, and (iv) nitrate reductase molybdenum cofactor assembly chaperone NarJ/NarW.

**Table 1:** DESeq2 analysis of differentially abundant microbial functions associated with metal tolerance and resistance (i.e., resistome) in the rhizobiome of *Noccaea praecox* when comparing metalliferous vs. non-metalliferous sites

KO	Function	log_2_FoldChange	padj	Ranking	Genes
K19976	Manganese transport system substrate-binding protein	−2.453	3.78e-03	Tolerance	*mntC*
K12957	Uncharacterized zinc-type alcohol dehydrogenase-like protein [EC:1.-. -.-].	−2.025	2.95e-03	Resistance	*yjgB*
K02188	Cobalt-precorrin-5B (C1)-methyltransferase [EC:2.1.1.195].	−1.534	4.79e-05	Resistance	*cbiD*
K00373	Nitrate reductase molybdenum cofactor assembly chaperone NarJ/NarW	−1.511	5.14e-04	Resistance	*narJ*, *narW*
K02009	Cobalt/nickel transport protein	1.159	6.39e-05	Tolerance	*cbiN*
K03893	Arsenical pump membrane protein	1.167	8.42e-03	Resistance	*arsB*
K15727	Membrane fusion protein, cobalt-zinc-cadmium efflux system	1.216	9.42e-10	Resistance	*czcB*
K08365	MerR family transcriptional regulator, mercuric resistance operon regulatory protein	1.340	9.55e-07	Resistance	*merR*
K15726	Cobalt-zinc-cadmium resistance protein CzcA	1.362	1.41e-18	Resistance	*czcA*
K11923	MerR family transcriptional regulator, copper efflux regulator	1.516	8.42e-03	Tolerance	*cueR*
K05795	Tellurium resistance protein TerD	1.558	2.70e-03	Resistance	*terD*
K11811	Arsenical resistance protein ArsH	1.618	5.99e-03	Resistance	*arsH*
K12956	Copper-transporting P-type ATPase V [EC:7.2.2.8].	1.716	8.42e-03	Resistance	*ctpV*
K15725	Outer membrane protein, cobalt-zinc-cadmium efflux system	2.620	2.54e-32	Tolerance	*czcC*, *cusC*, *cnrC*

Only statistically significant functions are listed in the table (*N* = 14; three and four samples for root and rhizosphere compartments of *N. praecox*, respectively, at each sampling site)

A comparison of roots and the rhizosphere at the metalliferous site identified 13 microbial functions that were differentially abundant and statistically significant (Table 2). In the root compartment, the most significantly increased functions included: (i) manganese-transporting P-type ATPase, (ii) cobalt/nickel-transporting P-type ATPase D, and (iii) molybdate transport system ATP-binding protein. On the contrary, the rhizosphere showed the enrichment in: (i) manganese/iron transport system ATP-binding protein, (ii) manganese/iron transport system permease protein, and (iii) manganese/iron transport system substrate-binding protein.

**Table 2:** DESeq2 analysis of differentially abundant microbial functions associated with metal tolerance and resistance (i.e., resistome) when comparing the root and rhizosphere compartments of *Noccaea praecox* from the metalliferous site

KO	Function	log2FoldChange	padj	Ranking	Genes
K09820	Manganese/iron transport system ATP-binding protein	−1.907	1.45e-04	Resistance	ABC.MN.A
K09819	Manganese/iron transport system permease protein	−1.679	4.40e-05	Resistance	ABC.MN.P
K09818	Manganese/iron transport system substrate-binding protein	−1.657	3.27e-03	Resistance	ABC.MN.S
K00947	Molybdenum storage protein	−1.407	9.10e-04	Resistance	*mosAB*
K11710	Manganese/zinc/iron transport system ATP- binding protein [EC:7.2.2.5]	−1.291	2.78e-04	Tolerance	*troB*, *mntB*, *znuC*
K07238	Zinc transporter, ZIP family	−1.076	4.40e-05	Tolerance	TC.ZIP, *zupT*, ZRT3, ZIP2
K02008	Cobalt/nickel transport system permease protein	−1.066	1.55e-04	Tolerance	*cbiQ*
K04652	Hydrogenase nickel incorporation protein HypB	−1.003	1.49e-03	Resistance	*hypB*
K13541	Cobalt-precorrin 5 A hydrolase/precorrin-3B C17-methyltransferase [EC:3.7.1.12 2.1.1.131]	1.103	4.69e-03	Resistance	*cbiGH*, *cobJ*
K11811	Arsenical resistance protein ArsH	1.612	5.41e-04	Resistance	*arsH*
K05776	Molybdate transport system ATP-binding protein	3.682	5.35e-08	Tolerance	*modF*
K12951	Cobalt/nickel-transporting P-type ATPase D [EC:3.6.3.-]	4.913	1.91e-03	Resistance	*ctpD*
K14950	Manganese-transporting P-type ATPase [EC:3.6.3.-]	8.571	7.50e-12	Resistance	ATP13A1

Only statistically significant functions are listed in the table (*N* = 7; three and four samples for root and rhizosphere compartments, respectively)

In contrast, at the non-metalliferous site, 23 microbial functions related to metal tolerance and resistance were found to differ significantly in abundance between the root and rhizosphere compartments (Table 3). Similar to results from the metalliferous site, root samples were enriched in manganese-transporting P-type ATPase and cobalt/nickel-transporting P-type ATPase D. In the rhizosphere, however, functions with significantly higher abundance included: (i) manganese transport system substrate-binding protein, (ii) manganese/iron transport system permease protein, and (iii) manganese/iron transport system substrate-binding protein.

**Table 3:** DESeq2 analysis of differentially abundant microbial functions associated with metal tolerance and resistance (i.e., resistome) when comparing the root and rhizosphere compartments of *Noccaea praecox* from the non-metalliferous site

KO	Function	log2FoldChange	padj	Ranking	Genes
K19976	Manganese transport system substrate-binding protein	−2.671	3.94e-07	Tolerance	*mntC*
K09819	Manganese/iron transport system permease protein	−2.215	1.35e-25	Resistance	ABC.MN.P
K09818	Manganese/iron transport system substrate-binding protein	−1.777	1.80e-04	Resistance	ABC.MN.S
K07238	Zinc transporter, ZIP family	−1.350	4.06e-10	Tolerance	TC.ZIP, *zupT*, ZRT3, ZIP2
K00373	Nitrate reductase molybdenum cofactor assembly chaperone NarJ/NarW	−1.298	3.36e-04	Resistance	*narJ*, *narW*
K02188	Cobalt-precorrin-5B (C1)-methyltransferase [EC:2.1.1.195].	−1.287	1.40e-09	Resistance	*cbiD*
K09817	Zinc transport system ATP-binding protein [EC:7.2.2.2.]	−1.123	2.51e-13	Tolerance	*znuC*
K09820	Manganese/iron transport system ATP-binding protein	−1.098	4.33e-04	Resistance	ABC.MN.A
K00520	Mercuric reductase [EC:1.16.1.1].	−1.072	1.11e-06	Resistance	*merA*
K19594	Gold/copper resistance efflux pump	1.024	4.43e-04	Tolerance	*gesB*, *mexQ*
K06324	Spore coat protein A, manganese oxidase [EC:1.16.3.3].	1.344	8.43e-06	Resistance	*cotA*
K16267	Zinc and cadmium transporter	1.408	1.06e-11	Resistance	*zipB*
K03893	Arsenical pump membrane protein	1.581	1.08e-04	Resistance	*arsB*
K11811	Arsenical resistance protein ArsH	1.690	2.41e-04	Resistance	*arsH*
K05795	Tellurium resistance protein TerD	2.100	1.59e-07	Resistance	*terD*
K07233	Copper resistance protein B	2.123	2.88e-04	Resistance	*pcoB*, *copB*
K05791	Tellurium resistance protein TerZ	2.130	2.17e-05	Resistance	*terZ*
K05792	Tellurite resistance protein TerA	2.382	3.25e-04	Resistance	*terA*
K05793	Tellurite resistance protein TerB	2.494	9.28e-03	Resistance	*terB*
K05776	Molybdate transport system ATP-binding protein	2.545	5.60e-03	Tolerance	*modF*
K12950	Manganese/zinc-transporting P-type ATPase C [EC:7.2.2.2.]	3.164	5.21e-03	Resistance	*ctpC*
K12951	Cobalt/nickel-transporting P-type ATPase D [EC:3.6.3.-]	6.963	7.40e-07	Resistance	*ctpD*
K14950	Manganese-transporting P-type ATPase [EC:3.6.3.-]	7.014	1.60e-07	Resistance	ATP13A1

Only statistically significant functions are listed in the table (*N* = 7; three and four samples for root and rhizosphere compartments, respectively)

## Discussion

In the present study, we observed that the location alone (metalliferous vs. non-metalliferous site) did not significantly influence the overall microbial functional traits, indicating its context-dependent effect. Also, the obtained results suggest functional redundancy within the microbiome that allows a stable core set of microbial functions to persist even under varying soil metal concentrations. In contrast, the soil compartment (i.e. roots vs. rhizosphere) was revealed to be a key factor shaping microbial functional traits of *N. praecox*, consistent with findings on hyperaccumulating *S. alfredii* [10]. The strong influence of soil compartment suggests that microbial colonization of *N. praecox* roots is not a stochastic process. The plant host likely exerts selective pressure to recruit microorganisms suited to this specific niche, consistent with PerMANOVA results showing no significant differences between the two locations. This was also observed in other studies, in which the plant host played a dominant role in shaping its root-associated microbiome [34, 52].

Comparison of KEGG level 2 functions revealed several groups that differed between the metalliferous and non-metalliferous site. These functions were related to metabolism, transposition or transfer of genetic material, transport, and environmental adaptations. They were more abundant at the metalliferous site, potentially contributing to metal resistance mechanisms. Similar observations were reported by several other studies that compared microbial functions in environments with different levels of metals [53–55]. As such, it can be a part of the general response of microbial soil communities to excess metals in the environment. To better understand how the metal concentrations and the root-associated microbiome influence specific microbial functions, we analyzed functions associated with metal tolerance and resistance, which could shape the accumulation and tolerance of the host plant.

### Genes associated with resistance and tolerance to metals

#### Effect of the increased concentration of soil metals

Several microbial resistome functions were affected by the habitat (metalliferous vs. non-metalliferous site), highlighting the strong influence of the environment in shaping microbial traits beyond the effects of the plant compartment alone. This confirms that abiotic factors, particularly elevated metal concentrations, exerted a strong selective pressure on microbial communities, influencing the presence and abundance of functions associated with metal tolerance and resistance.

The most abundant and statistically significant microbial functions distinguishing the two sites were associated with tolerance and resistance to Co/Zn/Cd and Te. These included the Co/Zn/Cd resistance protein CzcA (Co/Zn/Cd resistance), cobalt-zinc-cadmium efflux system (*czcC*, *cusC*, and *cnrC*), and the tellurite resistance protein TerC, which were significantly more abundant at the metalliferous than at the non-metalliferous site. The CzcCBA complex, to which *CzcA* and *CzcC* belong, is a membrane-bound protein system primarily found in Gram-negative bacteria, the dominant group in our samples [35]. This system is part of the resistance-nodulation-cell division (RND) family and mediates the efflux of divalent ions such as Co^2+^, Zn^2+,^ and Cd^2+^ from the cytoplasm and periplasm to the extracellular space [56–58]. CzcC protein acts as a modifier protein, extending the substrate specificity to Cd^2+^ and Co^2+^ [56, 57], and according to Rensing et al. (1997) [56], the deletion of the *czcC* gene results in a loss of resistance to these two ions. The high abundance of Co/Zn/Cd resistance functions at both sites likely reflects the constitutive Zn hyperaccumulation trait of *N. praecox* [3, 59]. However, extremely elevated concentrations of Zn and Cd at the metalliferous site (220 mg kg^− 1^ and 38 mg kg^− 1^, respectively), 22 and 34 times higher than at the non-metalliferous site [3], likely amplify this trend through direct environmental pressure.

Similar to proteins of the CzcCBA complex, the cobalt-zinc-cadmium efflux system (*czcC*, *cusC*, and *cnrC*) was significantly more abundant in the metalliferous site than at the non-metalliferous site. CusC, part of the protein efflux system CusCBA, provides bacteria resistance to Cu and Ag ions [60–62]. Additionally, the *cnrC* gene, along with *crnA* and *crnB*, forms a CnrCBA efflux complex which provides resistance to Co and Ni ions [63, 64]. Since Cu is an essential, yet potentially very toxic metal, strict regulation is required to prevent cell damage. The higher abundance of copper-transporting P-type ATPase V in the metalliferous site suggests that microbial communities are responding to a broader spectrum of metal stress. Since many bacterial resistance mechanisms are located on shared mobile genetic elements (MGE) [65], the higher abundance may reflect indirect selection pressure from other (toxic) elements, rather than Cu alone. A similar pattern was observed for Te; the increased abundance of the tellurite resistance protein TerC likely reflects co-selection driven by high concentrations of Cd, Zn, and Pb, rather than direct exposure to Te [66, 67]. Our findings highlight the selective pressure shaping microbial survival strategies in a former mining and smelting area where metal concentrations reach toxic levels. On the contrary, at the non-metalliferous site, microorganisms can invest energy in physiological processes that support growth and metabolism, rather than defence mechanisms.

#### Effect of the plant host

In addition to site-specific differences, the soil compartment (roots vs. rhizosphere) also shows distinct abundance patterns. Several microbial functions associated with the highly conserved ATP-binding cassette (ABC) transporter system were significantly more abundant in the rhizosphere compared to the root compartment, regardless of sampling site. These transporters can act as both exporters and importers of different molecules, including metals such as Mn, Fe, and Zn [68–70]. Transporters related to Mn/Fe were particularly abundant in the rhizosphere at both sampling sites. Mn and Fe are important nutrients for microorganisms, yet their bioavailability in soil is often limited due to their low solubility in neutral to alkaline soils [71, 72]. Soil pH in Lokovec and Žerjav was near neutral with values of 6.8 and 6.9, respectively [35], suggesting that microbes in the rhizosphere rely on high-affinity transport to acquire sufficient Mn and Fe. A significant abundance of Mn/Fe transporters likely reflects this need, especially in the rhizosphere, where intense plant-microbe and microbe-microbe competition drives nutrient acquisition strategies. Additionally, proteins from the ZnuABC, MntABC, and TroABCD were also significantly more abundant in the rhizosphere compared to the roots of *N. praecox* at both sampling sites. Several studies suggest that *znuABC* expression is repressed in cells with adequate Zn concentrations [73], and the high Zn affinity of the ZnuABC transporter plays a significant role in environments with scarce Zn or Zn availability [74]. As *N. praecox* is a Zn hyperaccumulator, the plant actively takes up large amounts of Zn, leaving less available Zn for microorganisms. Furthermore, upregulation of the *znuABC* genes may also lie in microbial competition for Zn, as it is one of the essential micronutrients for all living organisms [75–77]. Both MntABC and TroABCD have a high affinity to bind Mn. MntABC transporters are specific for Mn and Zn, occasionally also for Fe [78, 79], whereas TroABCD shows affinity primarily for Mn uptake [80]. Notably, the TroABCD manganese import system plays an important role in the oxidative stress of *Streptococcus suis* [80].

In addition, transporter proteins from the Zrt-/Irt-like protein (ZIP) family were also significantly more abundant in the rhizosphere compared to the roots, regardless of soil metal status. These proteins are particularly important in the metal uptake and homeostasis of essential nutrients like Zn, Fe, and Mn [81–83] and under metal deficiency, Cd can also be accumulated [82, 83]. In addition, the significant abundance of Zn importers in the rhizosphere could be due to the higher abundance of Zn-solubilising bacteria (ZnSB) in the rhizosphere of *N. praecox*, such as *Bacillus* sp [84, 85]. Taxa from *Bacillus* sp. were present only in the rhizosphere but not in the roots of *N. praecox* (Bočaj et al., unpublished results). Promoting ZnSB abundance in the rhizosphere could be beneficial for plants such as *N. praecox*, which has a high need for Zn uptake [86, 87]. Experiments with Zn and Cd hyperaccumulating *Arabidopsis helleri* demonstrated that intact soil microbial communities increased Cd uptake by 100% and Zn uptake by 15% compared to sterilized soils [88]. This underlines the strong impact of microorganisms on metal accumulation in hyperaccumulators. In the present study, we identified several groups of microbial transporters that can benefit *N. praecox* to satisfy its large appetite for Zn and could be an important part of its Zn hyperaccumulation mechanism.

The effect of the plant host was also evident in the root compartment. Transporting P-type ATPases, the molybdate transport system ATP-binding protein, and the arsenical resistance protein ArsH were significantly more abundant in the roots compared to the rhizosphere of *N. praecox*. This pattern was observed at both sampling sites. The Mn-transporting P-type ATPase (ATP13A1) is found in various fungi that also inhabit plant roots. For *Saccharomyces cerevisiae*, growth inhibition was evident at and above 30 mg Mn^− 1^ [89]. The significant abundance of Mn-transporting genes suggests that the root environment of *N. praecox* exerts selective pressure favouring microbes that can tolerate elevated Mn concentrations. Another P-type ATPase transporter (ATPase D) was significantly more abundant in the roots compared to the rhizosphere of *N. praecox* at both locations. This transporter, encoded by the *ctpD* gene, is associated with Co and Ni transport. Additionally, in the roots of *N. preacox* from the non-metalliferous site, a Mn/Zn-transporting P-type ATPase C was also detected. In *Mycobacterium smegmatis*, CtpD was shown to be important for Co and Ni homeostasis, whereas in *M. tuberculosis*, CtpD orthologs may be involved in metal detoxification by regulating intracellular concentrations of both metals [90]. On the other hand, in fungi, the same protein functions as a mitochondrial citrate transporter [91]. Citrate is an excellent chelating agent that binds and solubilizes metals [92, 93]. In addition, the Mo transport system ATP-binding protein (*modF* gene) was significantly more abundant in *N. praecox* roots compared to the rhizosphere at both sampling sites. Although its role is still unclear, it is likely involved in the transport and regulation of Mo [94, 95], which is an essential nutrient also for microorganisms that synthesize a wide range of metalloenzymes [96].

Moreover, As resistance functions, particularly the arsenical resistance protein ArsH, were significantly more abundant in the roots of *N. praecox* than in the rhizosphere. This trend was especially pronounced in the roots from the metalliferous site. However, ArsH is not only associated with As detoxification but also with resistance to chromate and ferric iron [97], and to organoarsenic compounds [98]. This suggests that the increased abundance of arsenic resistance functions may reflect exposure to a broader range of toxic elements. This is particularly evident in the root compartment, where oxidative stressors are likely more pronounced. In summary, the consistently higher abundance of specific microbial functions in the root compartment across both sampling sites strongly suggests that the root endosphere imposes distinct selective pressures. This environment, shaped by metal accumulation, plant immune responses, and oxidative stress, likely favours microbial taxa equipped with resistance and stress-adaptation mechanisms, independently of the surrounding soil conditions.

The consistently elevated abundance of several metal tolerance and resistance functions in *N. praecox* from both locations suggests a strong host-mediated effect on shaping the microbial resistome. This effect appears to override the influence of environmental pressures.

#### Plasmid-mediated metal resistance in the root-associated microbiome

In some cases, we observed enrichment of tolerance and resistance genes that cannot be explained by the metal soil concentration or plant selection (e.g., Cu, As, and Te). This is probably a result of a multimetal resistance/tolerance common in bacteria [28]. Multimetal resistance/tolerance genes in bacteria reside on plasmids such as pMOL28 and pMOL30, which usually co-occur and are frequently reported for Proteobacteria isolated from metalliferous environments [30]. As Gram-negative Proteobacteria were one of the most abundant phyla in both soil compartments and at both sites [35], this would explain the current observations. In addition, under selective pressure, these conjugative plasmids can be spread between bacteria, thus increasing the multi-metal resistance within microbial populations. Such transfer of metal-associated microbial functions could potentially contribute to the high proportion of Ni-tolerance reads in the resistome of *N. praecox*, although direct plasmid evidence was not assessed in our study. Apart from Cd, Zn, and Pb, some populations of *N. praecox* can also (hyper) accumulate Ni [99], with a more indicator-type of response [59, 100] and is not constitutive as in its closely related *N. goesingense* [101]. Therefore, we would not expect Ni-related resistance and tolerance to be profusely found in the resistome of *N. praecox* due to environmental pressure or host preference. Nevertheless, as mentioned, plasmids carry genes for the metals selected at the metalliferous site in our study (Cd, Zn, and Pb), as well as Ni-resistance/tolerance genes. These genes cannot be separated and are always found together. Although shotgun metagenomic sequencing offers a powerful insight into the composition and functions of microbial communities [102, 103], it remains limited by different methodological constraints like potential inaccuracies of the predictive functional profiling due to incomplete or biased reference databases, reduced resolution for rare or low-abundance taxa due to short-read lengths and uneven coverage, and technical artefacts such as contamination and extraction biases [103–105]. A combination of techniques, e.g., integrating metagenomic and metatranscriptomic data, could overcome these methodological shortcomings, leading to stronger interpretations of microbial functions under natural conditions.

Linking microbial functions to plant performance presents a challenge due to the complexity and context-dependence of plant–microbe interactions. Therefore, their functional traits often do not directly translate to activity under field conditions [106, 107]. Environmental factors, including soil type, moisture, and nutrient availability, can significantly influence both microbial metabolism and plant responses [108]. Additionally, many microbial functions are redundant across taxa, making it challenging to identify specific contributors to plant traits [109]. Nevertheless, an increasing number of papers report enhanced metal uptake and improved tolerance in hyperaccumulator plants after inoculation with beneficial microbes [110–113]. The effects were shown to be a result of increased solubilisation of metals by the microbes [110], improved nutrient uptake [111], upregulation of genes involved in metal transport and detoxification [112], and regulation of the oxidative stress response [113]. As microbial inoculations play a crucial role in enhancing the metal uptake and stress tolerance of hyperaccumulator plants, they represent a promising strategy for phytoremediation of contaminated soils. Nevertheless, formulation and specificity of plant-microbe interactions are critical points that need to be addressed for successful application [111]. As such, studies of hyperaccumulators in natural settings are an essential stepping stone, as they allow us to identify the most promising microbial inoculants for future experimental validations under controlled conditions. The relatively small number of metagenomic samples per site and their slight imbalance may reduce statistical power and affect analyses. Therefore, the results are interpreted conservatively, focusing on the most robust trends and acknowledging that some patterns may be over- or under-estimated.

## Conclusions

The results highlight the interplay between soil metal content and the plant host in shaping the microbial functional traits. Under metal-induced selective pressure that drives microbial adaptations through physiological flexibility and adaptive evolution of horizontal gene transfer, the plant host also plays a crucial, sometimes overriding role. Through its influence on the root-associated microbiome, the plant drives the enrichment of specific microbial functions. The combined pressures result in a dynamic and plastic microbial community shaped not only by environmental conditions but also by host-driven selection, reflecting the complex co-adaptation between hyperaccumulating plant and their microbiota. For hyperaccumulators such as *N. praecox*, this interaction may be essential for sustaining their unique lifestyle. The recruitment of microbes that facilitate metal uptake, detoxification, or transport would enhance the plant’s ability to thrive in metal-enriched soils. Understanding these plant-microbe interactions provides important insight into how hyperaccumulation is supported at the microbial level and may improve future strategies for phytoremediation or biofortification.

## Supplementary Information

Supplementary Material 1

## Data Availability

Plant sample identifiers and these data are available in the Herbarium of the University in Ljubljana under the identification LJU10147538.Differentially abundant microbial functions and these data are available in the Zenodo repository, 10.5281/zenodo.15639780. Co-occurrence networks are detailed under the Methods section, and raw datasets are available in the Zenodo repository, 10.5281/zenodo.15639780. Functional profiling data, Statistical analysis results, and these data are available under the Methods section, and raw datasets are available in the Zenodo repository, 10.5281/zenodo.15639780. Raw DNA-seq data have been deposited in the National Center for Biotechnology Information (NCBI) and can be accessed via the following link https://www.ncbi.nlm.nih.gov/bioproject/PRJNA1100267 under accessions: SRR28678510, SRR28678511, SRR28678526, SRR28678527, SRR28678528, SRR28678529, SRR28678530, SRR28678517, SRR28678518, SRR28678519, SRR28678520, SRR28678522, SRR28678523, SRR28678524.

## Abbreviations

Ag: Silver
As: Arsenic
Au: Gold
Cd: Cadmium
Co: Cobalt
Cr: Chromium
Cu: Copper
Fe: Iron
Hg: Mercury
HM: Heavy metals
KEGG: Kyoto Encyclopedia of Genes and Genomes
Mn: Manganese
Mo: Molybdenum
Ni: Nickel
Pb: Lead
ZIP: Zincregulated Ironregulated transporterlike Protein
Zn: Zinc
