# Supplementary material for: Microbial functional traits in the hyperaccumulating Noccaea praecox rhizobiome are metal-dependent and host-driven
Source: Environ Microbiome. 2026 Apr 5;21:70. doi: 10.1186/s40793-026-00890-y (PMC13188454; doi:10.1186/s40793-026-00890-y)
Supplement: Supplementary file 1 — Supplementary Material 1 [file 40793_2026_890_MOESM1_ESM.docx]

**Microbial functional traits in the hyperaccumulating *Noccaea praecox* rhizobiome are metal-dependent and host-driven**

Valentina Bočaj^1^, Paula Pongrac^1,2^, Matevž Likar^1^*

^1^University of Ljubljana, Biotechnical Faculty, Department of Biology, SI-1000 Ljubljana, Slovenia

^2^Jožef Stefan Institute, SI-1000 Ljubljana, Slovenia

*Correspondence: [matevz.likar@bf.uni-lj.si](mailto:matevz.likar@bf.uni-lj.si) (ORCID number 0000-0002-3086-6433)

[valentina.bocaj@bf.uni-lj.si](mailto:valentina.bocaj@bf.uni-lj.si) (ORCID number: 0009-0008-0309-2875)

[paula.pongrac@bf.uni-lj.si](mailto:paula.pongrac@bf.uni-lj.si) (ORCID number: 0000-0003-0721-7555)

**Supplementary material**

**Table S1** Microbial functions associated with metal tolerance and resistance in the root and rhizosphere compartments of *Noccaea praecox*. Each microbial function has a corresponding KO number from the KEGG database.

| **Ranking** | **KO** | **Metal** | **Function** | **Genes** | **Manual override** | |
| --- | --- | --- | --- | --- | --- | --- |
| **Resistance** | all KOs involved in chemical modification (reduction/oxidation/methylation), P-type ATPases, regulatory proteins specific to resistance operons (e.g., *zur*, *merR*), and those explicitly labelled 'resistance' in KEGG | | | | | |
|  | K03893 | As | arsenical_pump_membrane_protein | arsB | |  |
|  | K11811 | As | arsenical_resistance_protein_ArsH | arsH | |  |
|  | K02188 | Co | cobalt-precorrin-5B_(C1)-methyltransferase_[EC:2.1.1.195]. | cbiD | |  |
|  | K02230 | Co | cobalt_chelatase_CobN_[EC:6.6.1.2]. | cobN | |  |
|  | K03394 | Co | precorrin-2/cobalt-factor-2_C20-methyltransferase_[EC:2.1.1.130_2.1.1.151]. | cobI_cbiL | |  |
|  | K03795 | Co | sirohydrochlorin_cobalt_chelatase_[EC:4.99.1.3]. | cbiX | |  |
|  | K05895 | Co | precorrin-6A/cobalt-precorrin-6A_reductase_[EC:1.3.1.54_1.3.1.106]. | cobK_cbiJ | |  |
|  | K05936 | Co | precorrin-4/cobalt-precorrin-4_C11-methyltransferase_[EC:2.1.1.133_2.1.1.271]. | cobM_cbiF | |  |
|  | K06042 | Co | precorrin-8X/cobalt-precorrin-8_methylmutase_[EC:5.4.99.61_5.4.99.60]. | cobH_cbiC | |  |
|  | K09882 | Co | cobalt_chelatase_CobS_[EC:6.6.1.2]. | cobS | |  |
|  | K09883 | Co | cobalt_chelatase_CobT_[EC:6.6.1.2]. | cobT | |  |
|  | K13541 | Co | cobalt-precorrin_5A_hydrolase_/_precorrin-3B_C17-methyltransferase_[EC:3.7.1.12_2.1.1.131]. | cbiGH_cobJ | |  |
|  | K12951 | Co/Ni | cobalt/nickel-transporting_P-type_ATPase_D_[EC:3.6.3.-] | ctpD | |  |
|  | K15726 | Co/Zn/Cd | cobalt-zinc-cadmium_resistance_protein_CzcA | CzcCA | |  |
|  | K15727 | Co/Zn/Cd | membrane_fusion_protein,_cobalt-zinc-cadmium_efflux_system | czcB | |  |
|  | K07156 | Cu | copper_resistance_protein_C | copC_pcoC | |  |
|  | K07233 | Cu | copper_resistance_protein_B | pcoB_copB | |  |
|  | K07245 | Cu | copper_resistance_protein_D | pcoD | |  |
|  | K07665 | Cu | two-component_system,_OmpR_family,_copper_resistance_phosphate_ regulon_response_regulator_CusR | cusR_copR_silR | |  |
|  | K08344 | Cu | suppressor_for_copper-sensitivity_B | scsB | |  |
|  | K12956 | Cu | copper-transporting_P-type_ATPase_V_[EC:7.2.2.8]. | ctpV | |  |
|  | K17686 | Cu | P-type_Cu+_transporter_[EC:7.2.2.8]. | copA_ctpA_ATP7 | |  |
|  | K00520 | Hg | mercuric_reductase_[EC:1.16.1.1]. | merA | |  |
|  | K08363 | Hg | mercuric_ion_transport_protein | merT | |  |
|  | K08365 | Hg | MerR_family_transcriptional_regulator,_mercuric_resistance_operon_regulatory_ protein | merR | |  |
|  | K06324 | Mn | spore_coat_protein_A,_manganese_oxidase_[EC:1.16.3.3]. | cotA | |  |
|  | K11521 | Mn | two-component_system,_OmpR_family,_manganese_sensing_response_regulator | manR | |  |
|  | K14950 | Mn | manganese-transporting_P-type_ATPase_[EC:3.6.3.-] | ATP13A1 | |  |
|  | K15986 | Mn | manganese-dependent_inorganic_pyrophosphatase | ppaC | |  |
|  | K09818 | Mn/Fe | manganese/iron_transport_system_substrate-binding_protein | ABC.MN.S | | * |
|  | K09819 | Mn/Fe | manganese/iron_transport_system_permease_protein | ABC.MN.P | | * |
|  | K09820 | Mn/Fe | manganese/iron_transport_system_ATP-binding_protein | ABC.MN.A | | * |
|  | K12950 | Mn/Zn | manganese/zinc-transporting_P-type_ATPase_C_[EC:7.2.2.2.] | ctpC | |  |
|  | K00087 | Mo | xanthine_dehydrogenase_molybdenum-binding_subunit_[EC:1.17.1.4]. | ygeS_xdhA | |  |
|  | K00373 | Mo | nitrate_reductase_molybdenum_cofactor_assembly_chaperone_NarJ/NarW | narJ_narW | |  |
|  | K00947 | Mo | molybdenum_storage_protein | mosAB | |  |
|  | K03752 | Mo | molybdenum_cofactor_guanylyltransferase_[EC:2.7.7.77]. | mobA | |  |
|  | K07141 | Mo | molybdenum_cofactor_cytidylyltransferase_[EC:2.7.7.76]. | mocA | |  |
|  | K00518 | Ni | nickel_superoxide_dismutase_[EC:1.15.1.1]. | sodN | |  |
|  | K04652 | Ni | hydrogenase_nickel_incorporation_protein_HypB | hypB | |  |
|  | K07722 | Ni | CopG_family_transcriptional_regulator,_nickel-responsive_regulator | nikR | |  |
|  | K09121 | Ni | pyridinium-3,5-bisthiocarboxylic_acid_mononucleotide_nickel_chelatase_[EC:4.99.1.12]. | larC | |  |
|  | K11326 | Ni/Co | cation_efflux_system_protein_involved_in_nickel_and_cobalt_tolerance | nrsA_czcA | |  |
|  | K05791 | Te | tellurium_resistance_protein_TerZ | terZ | |  |
|  | K05792 | Te | tellurite_resistance_protein_TerA | terA | |  |
|  | K05793 | Te | tellurite_resistance_protein_TerB | terB | |  |
|  | K05794 | Te | tellurite_resistance_protein_TerC | terC | |  |
|  | K05795 | Te | tellurium_resistance_protein_TerD | terD | |  |
|  | K07803 | Zn | zinc_resistance-associated_protein | zraP | |  |
|  | K09823 | Zn | Fur_family_transcriptional_regulator,_zinc_uptake_regulator | zur | |  |
|  | K12957 | Zn | uncharacterized_zinc-type_alcohol_dehydrogenase-like_protein_[EC:1.-._-.-]. | yjgB | |  |
|  | K13979 | Zn | uncharacterized_zinc-type_alcohol_dehydrogenase-like_protein_[EC:1.-._-.-]. | yahK | |  |
|  | K02074 | Zn/Mn | zinc/manganese_transport_system_ATP-binding_protein | ABC.ZM.A | | * |
|  | K02075 | Zn/Mn | zinc/manganese_transport_system_permease_protein | ABC.ZM.P | | * |
|  | K02077 | Zn/Mn | zinc/manganese_transport_system_substrate-binding_protein | ABC.ZM.S | | * |
|  |  |  |  |  | |  |
| **Tolerance** | Tolerance: general transport systems (ABC transporters, permeases) and sequestration proteins | | |  | |  |
|  | K19594 | Au/Cu | gold/copper_resistance_efflux_pump | gesB_mexQ | |  |
|  | K19595 | Au/Cu | membrane_fusion_protein,_gold/copper_resistance_efflux_system | gesA_mexP | |  |
|  | K06189 | Co | magnesium_and_cobalt_transporter | corC | |  |
|  | K02006 | Co/Ni | cobalt/nickel_transport_system_ATP-binding_protein | cbiO | |  |
|  | K02007 | Co/Ni | cobalt/nickel_transport_system_permease_protein | cbiM | |  |
|  | K02008 | Co/Ni | cobalt/nickel_transport_system_permease_protein | cbiQ | |  |
|  | K02009 | Co/Ni | cobalt/nickel_transport_protein | cbiN | |  |
|  | K15725 | Co/Zn/Cd | outer_membrane_protein,_cobalt-zinc-cadmium_efflux_system | czcC_cusC_cnrC | |  |
|  | K16264 | Co/Zn/Cd | cobalt-zinc-cadmium_efflux_system_protein | czcD_zitB | |  |
|  | K07240 | Cr | chromate_transporter | chrA | |  |
|  | K07787 | Cu | copper/silver_efflux_system_protein | cusA_silA | |  |
|  | K07796 | Cu | outer_membrane_protein,_Cu(I)/Ag(I)_efflux_system | cusC_silC | |  |
|  | K07798 | Cu | membrane_fusion_protein,_Cu(I)/Ag(I)_efflux_system | cusB_silB | |  |
|  | K11923 | Cu | MerR_family_transcriptional_regulator,_copper_efflux_regulator | cueR | |  |
|  | K19591 | Cu | MerR_family_transcriptional_regulator,_copper_efflux_regulator | cueR | |  |
|  | K07810 | Cu/Ag | Cu(I)/Ag(I)_efflux_system_periplasmic_protein_CusF | cusF | |  |
|  | K02010 | Fe | iron(III)_transport_system_ATP-binding_protein_[EC:3.6.3.30] | afuC_fbpC | |  |
|  | K02011 | Fe | iron(III)_transport_system_permease_protein | afuB_fbpB | |  |
|  | K02012 | Fe | iron(III)_transport_system_substrate-binding_protein | afuA_fbpA | |  |
|  | K02013 | Fe | iron_complex_transport_system_ATP-binding_protein_[EC:3.6.3.34] | ABC.FEV.A | |  |
|  | K02015 | Fe | iron_complex_transport_system_permease_protein | ABC.FEV.P | |  |
|  | K02016 | Fe | iron_complex_transport_system_substrate-binding_protein | ABC.FEV.S | |  |
|  | K13283 | Fe | ferrous-iron_efflux_pump_FieF | fieF | |  |
|  | K11705 | Fe/Zn/Mn | iron/zinc/manganese/copper_transport_system_permease_protein | mtsC | |  |
|  | K07239 | HM | heavy-metal_exporter,_HME_family | TC.HME | |  |
|  | K03322 | Mn | manganese_transport_protein | mntH | |  |
|  | K11601 | Mn | manganese_transport_system_ATP-binding_protein_[EC:7.2.2.5] | mntA | |  |
|  | K11602 | Mn | manganese_transport_system_permease_protein | mntB | |  |
|  | K11603 | Mn | manganese_transport_system_substrate-binding_protein | mntC | |  |
|  | K19973 | Mn | manganese_transport_system_ATP-binding_protein_[EC:7.2.2.5] | mntA | |  |
|  | K19975 | Mn | manganese_transport_system_permease_protein | mntB | |  |
|  | K19976 | Mn | manganese_transport_system_substrate-binding_protein | mntC | |  |
|  | K11604 | Mn/Fe | manganese/iron_transport_system_substrate-binding_protein | sitA | |  |
|  | K11605 | Mn/Fe | manganese/iron_transport_system_permease_protein | sitC | |  |
|  | K11606 | Mn/Fe | manganese/iron_transport_system_permease_protein | sitD | |  |
|  | K11607 | Mn/Fe | manganese/iron_transport_system_ATP-binding_protein | sitB | |  |
|  | K11707 | Mn/Zn/Fe | manganese/zinc/iron_transport_system_substrate-binding_protein | troA_mntA_znuA | |  |
|  | K11708 | Mn/Zn/Fe | manganese/zinc/iron_transport_system_permease_protein | troC_mntC_znuB | |  |
|  | K11709 | Mn/Zn/Fe | manganese/zinc/iron_transport_system_permease_protein | troD_mntD_znuB | |  |
|  | K11710 | Mn/Zn/Fe | manganese/zinc/iron_transport_system_ATP-_binding_protein_[EC:7.2.2.5]. | troB_mntB_znuC | |  |
|  | K02017 | Mo | molybdate_transport_system_ATP-binding_protein_[EC:3.6.3.29] | modC | |  |
|  | K02018 | Mo | molybdate_transport_system_permease_protein | modB | |  |
|  | K02020 | Mo | molybdate_transport_system_substrate-binding_protein | modA | |  |
|  | K05776 | Mo | molybdate_transport_system_ATP-binding_protein | modF | |  |
|  | K11177 | Mo | xanthine_dehydrogenase_YagR_molybdenum-binding_subunit_[EC:1.17.1.4]. | yagR | |  |
|  | K02031 | Ni | peptide/nickel_transport_system_ATP-binding_protein | ddpD | |  |
|  | K02032 | Ni | peptide/nickel_transport_system_ATP-binding_protein | ddpF | |  |
|  | K02033 | Ni | peptide/nickel_transport_system_permease_protein | ABC.PE.P | |  |
|  | K02034 | Ni | peptide/nickel_transport_system_permease_protein | ABC.PE.P1 | |  |
|  | K02035 | Ni | peptide/nickel_transport_system_substrate-binding_protein | ABC.PE.S | |  |
|  | K10094 | Ni | nickel_transport_protein | cbiK | |  |
|  | K07238 | Zn | zinc_transporter,_ZIP_family | TC.ZIP_zupT_ZRT3_ZIP2 | |  |
|  | K07263 | Zn | zinc_protease_[EC:3.4.24.-]. | pqqL | |  |
|  | K08641 | Zn | zinc_D-Ala-D-Ala_dipeptidase_[EC:3.4.13.22]. | vanX | |  |
|  | K09815 | Zn | zinc_transport_system_substrate-binding_protein | znuA | |  |
|  | K09816 | Zn | zinc_transport_system_permease_protein | znuB | |  |
|  | K09817 | Zn | zinc_transport_system_ATP-binding_protein_[EC:7.2.2.2.] | znuC | |  |
|  | K16922 | Zn | putative_peptide_zinc_metalloprotease_protein | yydH | |  |
|  | K16267 | Zn/Cd | zinc_and_cadmium_transporter | zipB | |  |

* Literature identifies these specific uptake systems (often corresponding to sitABCD or znuABC families) not merely as housekeeping nutrient importers, but as high-affinity scavenging pumps. They are critical for "nutritional immunity" and survival in metal-depleted environments created by host defenses. This active, high-specificity capability functions as a survival mechanism parallel to detoxification, distinguishing them from passive or general nutrient transport systems.

**Table S2** Statistically significant differences (padj < 0.01) in Wilcoxon test for KEGG level 2 functions in the root (N=6) and rhizosphere (N=8) compartments of *N. praecox* between the non-metalliferous (Lokovec) and metalliferous (Žerjav) sites.

| KEGG level 2 | p | padj | mean_Lokovec | mean_Žerjav |
| --- | --- | --- | --- | --- |
| Folding, sorting and degradation | 4.74e-05 | 5.05e-04 | 466 | 609 |
| Protein families: genetic information processing | 3.09e-11 | 9.89e-10 | 352 | 411 |
| Protein families: metabolism | 3.40e-03 | 1.40e-02 | 290 | 325 |
| Signal transduction | 3.49e-03 | 1.40e-02 | 390 | 415 |
| Transcription | 7.01e-04 | 4.48e-03 | 251 | 304 |
| Unclassified: genetic information processing | 4.73e-03 | 1.68e-02 | 1,096 | 702 |
| Environmental adaptation | 3.31e-03 | 1.40e-02 | 76.9 | 250 |
| Metabolism of terpenoids and polyketides | 3.18e-04 | 2.54e-03 | 194 | 198 |
| Transport and catabolism | 4.57e-06 | 7.32e-05 | 37.1 | 103 |

**Table S3** General PerMANOVA for microbial functions in the root (N=6) and rhizosphere (N=8) compartments of *N. praecox* from the non-metalliferous and metalliferous locations.

| **Factor** | **Df** | **SumOfSqs** | **R2** | **F** | **Pr(>F)** |
| --- | --- | --- | --- | --- | --- |
| soil_compartment | 1 | 0.011781368 | 0.25694808 | 6.620942 | 0.003 |
| location | 1 | 0.003565662 | 0.07776601 | 2.003845 | 0.127 |
| soil_compartment:location | 1 | 0.009151218 | 0.19958530 | 5.142840 | 0.008 |
| Residual | 12 | 0.021352914 | 0.46570062 |  |  |
| Total | 15 | 0.045851161 | 1.00000000 |  |  |

**Table S4** General PerMANOVA for microbial resistome in the root (N=6) and rhizosphere (N=8) compartments of *N. praecox* from the non-metalliferous and metalliferous locations.

| **Factor** | **Df** | **SumOfSqs** | **R2** | **F** | **Pr(>F)** |
| --- | --- | --- | --- | --- | --- |
| soil_compartment | 1 | 0.022 | 0.269 | 11.023 | 0.0002 |
| location | 1 | 0.033 | 0.406 | 16.633 | 0.0001 |
| soil_compartment:location | 1 | 0.006 | 0.080 | 3.278 | 0.0343 |
| Residual | 10 | 0.020 | 0.244 | N/A |  |
| Total | 13 | 0.080 | 1.000 | N/A |  |
